# Phenotypic Association Analyses With Copy Number Variation in Recurrent Depressive Disorder

**DOI:** 10.1016/j.biopsych.2015.02.025

**Published:** 2016-02-15

**Authors:** James J.H. Rucker, Katherine E. Tansey, Margarita Rivera, Dalila Pinto, Sarah Cohen-Woods, Rudolf Uher, Katherine J. Aitchison, Nick Craddock, Michael J. Owen, Lisa Jones, Ian Jones, Ania Korszun, Michael R. Barnes, Martin Preisig, Ole Mors, Wolfgang Maier, John Rice, Marcella Rietschel, Florian Holsboer, Anne E. Farmer, Ian W. Craig, Stephen W. Scherer, Peter McGuffin, Gerome Breen

**Affiliations:** a Medical Research Council Social Genetic and Developmental Psychiatry Centre, Institute of Psychiatry, King’s College London, London, United Kingdom; b The Centre for Applied Genomics and Program in Genetics and Genomic Biology, The Hospital for Sick Children, Toronto, Ontario, Canada; c Medical Research Council Centre for Neuropsychiatric Genetics and Genomics, Neuroscience and Mental Health Research Institute, Cardiff University, Cardiff, Wales, United Kingdom; d GlaxoSmithKline, Verona, Italy, and Greenford; e National Institute for Health Research Biomedical Research Centre, South London and Maudsley National Health Service Foundation Trust and Institute of Psychiatry, King’s College London; f Barts and The London School of Medicine and Denistry, Queen Mary University of London, London, United Kingdom; g Department of Psychiatry, University Hospital of Lausanne, Lausanne, Switzerland; h Department of Psychiatry, Dalhousie University, Halifax, Nova Scotia, Canada; i Department of Psychiatry, University of Bonn, Bonn, Germany; j Department of Psychiatry, Washington University, St Louis, Missouri; k Central Institute of Mental Health, Mannheim; l Max Planck Institute of Psychiatry (FH), Munich, Germany; m Centre of Psychiatric Research, Aarhus University Hospital, Risskov, Denmark; n Department of Psychiatry, University of Alberta, Edmonton, Alberta, Canada; o Department of Psychiatry, University of Birmingham, Birmingham, United Kingdom; p Section of Psychiatry, Institute of Neurosciences, Biomedical Research Centre, CIBERSAM, University of Granada, Granada, Spain; q Departments of Psychiatry and Genetics and Genomic Sciences, Seaver Autism Center, The Mindich Child Health & Development Institute, Mount Sinai School of Medicine, New York, New York; r McLaughlin Centre and Department of Molecular Genetics, University of Toronto, Toronto, Ontario, Canada

**Keywords:** Affective disorders, Copy number variation, Depression, Genetics, Phenotypes

## Abstract

**Background:**

Defining the molecular genomic basis of the likelihood of developing depressive disorder is a considerable challenge. We previously associated rare, exonic deletion copy number variants (CNV) with recurrent depressive disorder (RDD). Sex chromosome abnormalities also have been observed to co-occur with RDD.

**Methods:**

In this reanalysis of our RDD dataset (*N* = 3106 cases; 459 screened control samples and 2699 population control samples), we further investigated the role of larger CNVs and chromosomal abnormalities in RDD and performed association analyses with clinical data derived from this dataset.

**Results:**

We found an enrichment of Turner’s syndrome among cases of depression compared with the frequency observed in a large population sample (*N* = 34,910) of live-born infants collected in Denmark (two-sided *p* = .023, odds ratio = 7.76 [95% confidence interval = 1.79–33.6]), a case of diploid/triploid mosaicism, and several cases of uniparental isodisomy. In contrast to our previous analysis, large deletion CNVs were no more frequent in cases than control samples, although deletion CNVs in cases contained more genes than control samples (two-sided *p* = .0002).

**Conclusions:**

After statistical correction for multiple comparisons, our data do not support a substantial role for CNVs in RDD, although (as has been observed in similar samples) occasional cases may harbor large variants with etiological significance. Genetic pleiotropy and sample heterogeneity suggest that very large sample sizes are required to study conclusively the role of genetic variation in mood disorders.

Recurrent major depressive disorder (RDD) is associated with high morbidity, high economic burden, and high rates of suicide (1, 2, 3, 4). The genomic basis of the likelihood of developing RDD is largely unknown (5). Twin studies suggested that recurrent and severe forms of major depressive disorder are particularly heritable forms (6, 7). However, genome-wide association studies with single nucleotide polymorphisms showed inconsistent results (8, 9), and a mega-analysis failed to find any genome-wide significant associations (10), suggesting that other forms of genetic variation may be responsible for the observed heritability. Large, rare copy number variants (CNVs), defined as deletions or duplications of genomic material > 1000 base pairs in length, have been identified and associated with a range of psychiatric disorders (11, 12, 13, 14, 15), although the evidence for association with mood disorders is unclear (16, 17, 18, 19, 20). We previously showed an enrichment of rare, exonic deletion CNVs in a sample of RDD, with a low frequency of such variants in a screened control sample (20). Large chromosomal abnormalities are also easily detectable by DNA microarray (21). Various abnormalities, particularly sex chromosome aneuploidies (22, 23) and the 22q11.2 microdeletion (24), have been associated with psychiatric diagnoses such as schizophrenia and mood disorders, although association with any specific phenotype is usually incomplete (25).

We investigated the frequency of sex chromosome aneuploidies and large, rare CNVs in 3106 cases of RDD. We hypothesized that chromosomal aneuploidies, large (>100 kb) rare CNVs, and particularly CNVs located in regions previously associated with psychiatric disorders may be associated with a diagnosis of RDD; a younger age of onset; longer duration of illness; and measures of neuroticism, psychoticism, and extraversion made in our dataset. We compared our case sample with a control sample screened for a lifetime absence of psychiatric disorder (*n* = 459) and an unscreened population control sample (*n* = 2699) from phase 2 of the Wellcome Trust Case Control Consortium (WTCCC2) and, for sex chromosome aneuploidies only, with data from karyotype analysis undertaken in 34,910 sequentially screened live-born infants in Denmark reported by Nielsen and Wohlert (26).

## Methods and Materials

### Samples

Samples comprised 3106 cases (2197 female and 909 male) taken from three studies of RDD: Genome Based Therapeutic Drugs for Depression (27), Depression Network study (28), and Depression Case Control study (29). This sample set is almost identical to the sample set analyzed in our previous work (20); however, calling methods and quality control procedures have been updated and varied according to the length of CNV being called. Further details of the contributing studies are provided in Supplement 1. All samples were derived from venous blood collected at the time of interview and extracted in the same laboratory. All samples are from individuals with European origin. Informed written consent was obtained from all participants, and all studies were approved by relevant local ethics committees. As an additional control set, we used 2699 control samples (1354 female and 1345 male) run on Infinium 1M bead arrays (Illumina, Inc., San Diego, California) from phase 2 of the WTCCC2 representing the National Blood Service cohort, derived from subjects who donated blood to the United Kingdom blood services collection.

### Phenotypic Data Collection and Extraction

The phenotypic data from across studies included in this dataset were previously integrated into a single database (30). We extracted data on the following items: 1) age at first onset of disorder, 2) duration of worst episode, 3) trait neuroticism scores, 4) trait psychoticism scores, and 5) trait extraversion scores. Trait personality scores are derived from the Eysenck Personality Questionnaire (31). See Supplement 1 for more details.

### Genotyping

Samples were genotyped on the HumanHap 610-Quad Beadchip (Illumina, Inc.) and contemporaneously processed at the same laboratory. Raw probe intensity data were processed according to the manufacturer’s guidelines with the GenomeStudio platform (Illumina, Inc.) to obtain the normalized probe intensity at each marker and the log R ratio and B allele frequency at each marker.

### CNV Calling

To make CNV calls, we processed fluorescence intensity data for autosomal markers common to each Illumina array (*n* = 562,680) using three separate algorithms: PennCNV (32) (version released August 2009); QuantiSNP v2.3 (33), and iPattern (34) in liaison with the authors.

### Sample and CNV Quality Control

We analyzed all samples for chromosomal aneuploidies because they are rare and pragmatic to confirm visually. We used measures of the heterozygosity of the B allele frequency, calculated by PennCNV for chromosome X, and the mean of the log R ratio of chromosome Y, calculated in R (35), to make two predictions of gender for each sample and then looked for discordances between the two predictions. In addition to comparing the frequency of sex chromosome aneuploidy in our case and control sets, we also compared the frequency detected in 34,910 sequentially screened live-born infants from an observational study by Nielsen and Wohlert (26).

To detect autosomal aneuploidy, we calculated the log R ratio mean and B allele frequency heterozygosity for each chromosome and visually inspected plots where this value deviated by >3 SD from the mean for the value taken across all autosomes in that sample. For phenotypic association analyses, we used sample-wide quality control metrics calculated by Genome Studio and the PennCNV algorithm as well as additional metrics calculated with code derived from the CNVision package (36). For detailed methods, including specific thresholds and CNV merging definitions, see Supplement 1.

### CNV Validation

To validate a subset of our CNV calls, we used a customized high-density oligonucleotide comparative genomic hybridization array (4×180K, Agilent Technologies, Santa Clara, California) in liaison with Oxford Gene Technologies (Oxfordshire, United Kingdom). We analyzed 183 samples from our cases and screened control samples on the comparative genomic hybridization array. Of 183 samples, 36 CNVs in 35 samples were available for follow-up. All 36 CNVs (100%) were validated. Full details of the regions used for follow-up, CNVs available for validation, and further information regarding array comparative genomic hybridization laboratory protocols are provided in Supplement 1.

### Power Calculations

Power calculations in CNV studies are problematic because effect sizes and models of association are based on approximations that may be unrealistic. For comparisons of sex chromosome abnormalities, a post hoc calculation of power given our figures for Turner’s syndrome suggests we have a 99% power to detect a significant effect. For analyses of large CNVs, assuming a threefold enrichment between cases and control samples, we have 75% power to detect an effect. For phenotypic analyses, on the assumption of a linear model of association with phenotype, a rare variant effect size of .005, and a type 1 error probability of .05, we calculate that our sample size has 88% power to detect an effect from rare CNVs occurring over the whole genome.

### Statistical Analysis

Frequencies of samples with large CNVs and aneuploidies were compared with Fisher’s exact test (*p* values for one-sided and two-sided tests are shown). Whole-genome analyses of CNV burden between cohorts were performed using permutation analysis in PLINK v1.07 (37). An initial α level of .05 was set. Burden analyses were divided into three sets of tests—cases versus all control samples, cases versus screened control samples, and cases versus WTCCC2 control samples. Each of these sets of larger analyses was subdivided further into analyses considering all CNVs, deletion CNVs, and duplication CNVs. Within these subsets of analysis, 7 individual burden tests were performed, resulting in a total of 63 burden tests (Supplement 1). We set a Bonferroni corrected significance *p* value of .00079. This significance level is likely to be conservative because the datasets are not independent. For phenotypic analysis, we set an initial α level of .05, then used matrix spectral decomposition of the correlation matrix between the five phenotypic variables analyzed (38), implemented at http://gump.qimr.edu.au/general/daleN/matSpD/, resulting in a corrected α level of .0073. Statistical association with phenotypic data was performed using linear regression implemented in STATA/IC v10.1 (39). Power calculations were performed with G*Power v3.1.7 and the pwr package (version 1.1.1), implemented in R (35).

## Results

### Sex Chromosome and Autosome Aneuploidies

All 3106 case samples were analyzed for sex chromosome aneuploidy. Of 2197 female cases, we detected 3 cases of 45,X (Turner’s syndrome) (Figure S2A in Supplement 1), of which 2 were probable 45,X/46,XX mosaics (Figure S2B in Supplement 1). Of 909 male cases, we detected 2 cases of 47,XXY (Klinefelter’s syndrome), one of which had an additional deletion of Yq (Figure S3 in Supplement 1). Significantly more cases of 45,X were observed in our case sample compared with the population sample (*N* = 34,910) of live-born infants reported by Nielsen and Wohlert (26) (two-sided *p* = .023, odds ratio [OR] = 7.76 [95% confidence interval (CI) = 1.79–33.6]). No other comparisons were statistically significant. One case of 47,XXY (Klinefelter’s syndrome) was detected in the WTCCC2 control samples. Full results are presented in Table 1.

We detected no autosomal aneuploidies. We detected one case of diploid/triploid mosaicism (Figure S4 in Supplement 1). Three cases were found to harbor complete uniparental isodisomy (UPD) for a single chromosome (one in chromosome 12 [Figure S5A in Supplement 1] and two in chromosome 4 [Figure S5B, C in Supplement 1]). Two samples from the WTCCC2 control group were also found to harbor UPD of a whole chromosome (one of chromosome 13 [Figure S5D in Supplement 1] and one of chromosome 21 [Figure S5E in Supplement 1]) (one-sided *p* = .57, two-sided *p* = 1.00, OR = 1.30 [95% CI = .26–∞). No samples from the screened control group showed UPD (one-sided *p* = .66, two-sided *p* = 1.00).

### Large CNVs

Large CNVs are defined as having length >1 Mb and called with >100 markers. All CNV details are published in Table S3 in Supplement 1. Large CNV frequencies are shown in Table 2.

Of 6264 samples, 5430 passed sample quality control (2723 case samples, 348 screened control samples, and 2359 WTCCC2 control samples). Among 123 (2.3%) samples, 126 CNVs >1 Mb were detected. Of these CNVs, 36 (28.5%) were heterozygous deletions, and 90 (71.5%) were heterozygous duplications. Size of CNVs was as follows: 3 (2.4%) were >5 Mb, 2 (1.6%) were between 3 Mb and 5 Mb, 11 (8.7%) were between 2 Mb and 3 Mb, and the rest (*n* = 110; 87.3%) were between 1 Mb and 2 Mb.

We detected 77 CNVs in 74 of 2723 case samples. We detected 4 CNVs in 4 of 348 screened control samples (two-sided *p* = .10, OR = 2.40 [95% CI = .91–6.36]). Stratifying by CNV type, 21 deletion CNVs were observed in 20 cases, and 2 deletion CNVs were observed in 2 control samples (two-sided *p* = 1.00, OR = 1.28 [95% CI = .33–NaN]). There were 56 duplication CNVs observed in 54 cases, whereas 2 duplication CNVs were observed in 2 control samples (two-sided *p* = .084, OR = 3.46 [95% CI = .93–NaN]).

Of 2359 WTCCC2 control samples, we detected 45 CNVs in 45 samples. As stated earlier, 77 CNVs were seen in 74 case samples (two-sided *p* = .063, OR = 1.44 [95% CI = .99–2.09]). Stratifying by CNV type, 21 deletion CNVs from 20 cases were observed in our case sample, whereas in the WTCCC2 control sample, 13 deletion CNVs were observed in 13 samples (two-sided *p* = .49, OR = 1.34 [95% CI = .67–2.65]). There were 56 duplication CNVs observed in 54 cases, whereas 32 duplication CNVs were observed in 32 WTCCC2 control samples (two-sided *p* = .10, OR = 1.45 [95% CI = .94–2.25]).

In 141 cases (37 male and 104 female), mood-congruent psychotic symptoms were demonstrated. There were five CNVs in five case (two male and three female) within this subset. We compared these data with CNV frequencies in our screened control sample, in which a history, family history, or current presentation of psychosis were exclusion criteria. Of 348 control samples, we detected four CNVs in 4 samples (two-sided *p* = .13, OR = 3.16 [95% CI = .90–11.04]). We stratified for deletions, where none were observed in the psychotic cases group and two CNVs in two samples were observed in the screened control group (two-sided *p* = .51, OR = 0 [95% CI = 0–4.75]), and duplications, where five CNVs in five cases were observed in the psychotic cases group and two CNVs in two samples were observed in the screened control group (two-sided *p* = .023, OR = 6.36 [95% CI = 1.40–∞]).

Several very large variants were observed in this dataset. See Supplement 1 for further details.

### Phenotypic Association Analyses

To minimize the effect of false-positive calls clustering in different sample groups, CNVs >100 kb, called with at least 10 markers, in high-quality samples and called using three algorithms were considered in this analysis. Of 3106 samples, 2397 cases (77.2%) (717 male and 1680 female) passed sample quality control. Phenotypic data were available in 1940 (80.1%) of these samples. Data from the Eysenck Personality Trait questionnaire were unavailable in the GENDEP study, which limited our analysis in these instances to a sample of 1631.

There were 1337 rare CNVs detected, of which 648 (48.5%) were deletion CNVs and 689 (51.5%) were duplication CNVs. Of CNVs, 106 fell within regions previously associated with schizophrenia; 36 (34.0%) were deletion CNVs, and 70 (66.0%) were duplication CNVs. There were 402 CNVs defined as singleton events (i.e., occurring only once in the dataset); 205 (41.7%) CNVs were deletions, and 287 (58.3%) were duplications. Results for whole-genome CNV association analyses with phenotypes are shown in Table S4 in Supplement 1, analyses of singleton CNVs are shown in Table S5 in Supplement 1, and analyses restricted to CNVs within previously associated regions of the genome are shown in Table S6 in Supplement 1.

Age of onset data were available in 1926 samples (98.7%). No significant association was found between global rare CNV burden per sample and age of onset of disorder. A nonsignificant trend was found between the number of rare CNVs falling over regions previously associated with schizophrenia and increased age of onset of disorder (*t* = 2.12, *p* > |*t*| = .03) (Table 3). Reanalyzing by region, this association was driven by duplications in 15q13.3 (*t* = 1.9, *p* > |*t*| = .06) (Table S9 in Supplement 1). No significant association was found between singleton CNV burden and age of onset of disorder.

Duration of worst episode data were available in 977 samples (50.3%). No significant associations were found between measures of rare CNV burden and duration of worst episode.

Eysenck Personality Questionnaire data were available in 1631 samples. Trait neuroticism data were available in 1580 samples (96.9%), trait psychoticism data were available in 1619 samples (99.3%), and trait extraversion scores were available in 1619 samples (99.3%). No significant associations were found in any analyses between personality trait scores and CNV burden.

### Whole-Genome Burden Analysis

This dataset is similar to the dataset used for our previous research (20), in which we showed evidence that samples with genic deletion CNVs >100 kb in size were more common in RDD cases than control samples. We decided to revisit this hypothesis using this dataset, which is subject to more stringent quality control parameters and uses three algorithms to call CNVs, rather than one. Of samples, 2397 case samples, 332 screened control samples, and 2151 WTCCC2 control samples passed sample quality control. Compared with our original results, we found no evidence to support the notion that CNVs are more common in RDD cases, but deletion CNVs in cases tended to harbor more genes in RDD cases than control samples. Full results are shown in Table S13 in Supplement 1. Briefly, although there were no significant differences between the proportion of samples that harbored a deletion CNV (two-sided *p* = .55), the deletion CNVs that were seen contained significantly more genes in case samples than in control samples when control cohorts were combined (two-sided *p* = .0002) as well as independently and in the same graduated manner observed in our previous analysis. A trend suggesting that duplication events were more common in control samples was seen (two-sided *p* = .049); however, the absolute difference was modest.

## Discussion

We present an analysis of large CNVs and chromosomal aneuploidies in a case-control sample of RDD. After correction for multiple testing, we generally found little evidence for the association of large CNVs with RDD. We found an enrichment of undiagnosed Turner’s syndrome in RDD cases compared with a large population sample (*N* = 34,910) of live-born infants in Denmark. We found further examples of other sex chromosome abnormalities and autosomal UPD in the case sample. We also present an analysis of global CNV burden. In a reanalysis of rare CNVs >100 kb, there was little evidence of differences in major measures of CNV burden; however, the number of genes falling within deletion CNVs seen was significantly higher in cases than in both control groups, even after correction for multiple testing. When CNVs >1 Mb in size were considered in isolation, we found they occurred more frequently in the case sample compared with the screened control sample and with the WTCCC2 control sample, but this difference fell short of statistical significance. The increase in frequency was driven by large duplication CNVs. A further association analysis with phenotypic measures found a nonsignificant trend between the burden of duplication events over regions previously associated with schizophrenia and increased age of onset. This trend was driven by duplications in the region 15q13.3. Usually CNVs of this size are rare, and we may lack power to detect an effect. An aggregated association analysis of CNVs is also hampered by pleiotropy in the regions studied; however, without a greatly increased sample size, analyzing by region is invariably underpowered. We present both methods of analysis here.

Some evidence exists for the enrichment of sex chromosome aneuploidies in psychiatric disorders. One of the largest studies was published by Maclean *et al.* in 1968 (40), where the buccal smear method (41) was used to distinguish between cells with chromatin bodies of various numbers in the different sexes. This technique does not reliably detect Turner’s syndrome because it relies on the process of X inactivation to generate visible chromatin bodies. Nonetheless, this study found a significant enrichment of sex chromosome abnormalities among patients in psychiatric hospitals at the time compared with a control population. However, the inclusion of cases of schizophrenia, “mental deficiency,” and “epileptic insanity” suggests a sample qualitatively different from our own. Mood disorders are known to be common in people with sex chromosome abnormalities (22), and the relative enrichment of cases of Turner’s syndrome in our sample is unsurprising. The standardized mortality ratio for patients with Turner’s syndrome who survive infancy is estimated to be 3.6 (42), and the use of a live-born sample compared with a sample of adults is likely to underestimate the true difference. It is possible that the cases of mosaic 45,X/46,XX observed are derived from somatically acquired 45,X cells seen solely in blood. Overall, our results add evidence to the association of Turner’s syndrome with RDD. There is little evidence for the association of Klinefelter’s syndrome with mood disorders (22), and we found no evidence of this, although our sample probably lacks sufficient power (54.6% assuming a twofold enrichment and a base population frequency of 1:1000).

The detection of a diploid/triploid mosaic is notable. Previously rare, DNA microarray studies have already documented this phenomenon (43, 44). Individuals with congenital diploid/triploid mosaicism tend to be profoundly disabled (44, 45) and are unlikely to have been included in our study. This variant is probably a somatically acquired abnormality.

Five instances of UPD—in chromosome 4 (two cases), chromosome 12 (one case), chromosome 13 (one WTCCC2 control), and chromosome 21 (one WTCCC2 control)—were noted. When an individual receives two identical copies of a chromosome, or part of a chromosome, from one parent, it is known as UPD. All five instances of UPD observed in this sample set had log R ratio values indicating diploid copy number, and four of five demonstrated complete loss of heterozygosity across the chromosome. This finding suggests the mechanism of monosomy rescue (by which chromosomal monosomy is avoided by duplication of the remaining chromosome during gametogenesis) and an origin in meiosis II (43). One sample demonstrated a resumption of heterozygosity at the distal end of chromosome 21q, suggesting that partial recombination has occurred and an origin later in meiosis II (43). No significant differences in frequency were observed between the case and control samples, although our study may have been underpowered to detect an effect. The significance of these observations is unclear, although UPD is expected to disrupt imprinting and increase the chance of homozygosity for a recessive mutation (46).

In our previous analysis, we showed that rare deletion CNVs >100 kb in length were significantly associated with our case sample, with a particularly low frequency of deletions being seen in our screened control sample (20). This study could be criticized for using only one calling algorithm for identification of CNVs between 100 kb and 1 Mb. We reanalyzed this dataset using more stringent quality control parameters and three algorithms for CNV detection because more recent research has shown that CNV algorithms are subject to high type 1 error rates (47, 48, 49, 50, 51, 52). Our reanalysis indicated that although the proportion of samples containing CNVs is not significantly different between cases and control samples, deletion CNVs within case samples encompass significantly more genes than control samples. It could be argued that restricting analysis to calls made by three algorithms is overly conservative, resulting in type 2 calling errors that reduce power to detect association. This dichotomy illustrates the tricky balance to be struck between known type 1 errors and unknown type 2 errors in CNV calling, which is likely to affect all analyses relying on indirect measures of genomic copy number.

In an attempt to clarify the relationship of CNV burden with RDD, we analyzed the relationship between CNV and phenotype using clinical measures taken during sample ascertainment. In general, no statistically significant associations were observed. A trend was observed between CNVs occurring over regions previously associated with schizophrenia and increased age of onset. This trend was driven by duplication events in the 15q13.3 region. The CNVs in this region all encompass the gene encoding the alpha 7 nicotinic cholinergic receptor (*CHRNA7*). This CNV has also been implicated in Alzheimer’s disease (53), and a recent study (using some samples also included in this work) found that this duplication was associated with a poorer response to antidepressant medication (54). Although the relevance of this observation to RDD is unclear, the occurrence of this duplication in association with age of onset in the context of other research implicating it in dementia and poorer response to antidepressants is notable, especially given the clinical crossover between depression and dementia observed in older adults (55). A full table of results and illustrations of the CNVs in these areas can be found in Supplement 1.

A significant limitation of this study is the small sample size of screened control samples. The small sample size occurred because most subjects in this cohort contributed DNA via cheek swab, which we found to be of insufficient quality to call CNVs reliably. Although our case sample is large, it is probably of insufficient size to determine definitively the role of this level of genomic variation in this clinical group.

In conclusion, this study adds little evidence to the notion that rare CNVs are associated with RDD (in contrast to our previous analysis), although deletion CNVs that do occur in this group were shown to harbor more genes than deletion CNVs occurring in control samples. This may be a relevant finding because deletion CNVs, with concomitant loss of function, are expected to be more deleterious than duplications. Occasional large CNVs and chromosomal aneuploidies are seen in isolated cases. We found no evidence to suggest that duration of worst episode and personality traits as measured by the Eysenck Personality Questionnaire are associated with rare CNVs in cases of RDD, and other trends for significance fall short after correction for multiple testing. In general, the evidence for the involvement of CNVs in cases of mood disorders appears much less convincing than the evidence in cases of autism and schizophrenia. Genetic pleiotropy and sample heterogeneity in mood disorder samples as well as the equivocal results from current studies suggest that much larger sample sizes are required to determine conclusively whether this level of genomic variation is of relevance. Further light may be shed on this issue by CNV meta-analyses from worldwide collaborations of large sample sets, particularly the psychiatric genome-wide association studies consortium (56).

## Figures and Tables

**Table 1:** Frequency of Sex Chromosome Aneuploidy in Cases,^a^ Population Control Samples, WTCCC2 Control Samples, and Screened Control Samples

Sex	Cases		Population Control Samples		WTCCC2 Control Samples		Screened Control Samples	
Female	Male	Female	Male	Female	Male	Female	Male
Clinical Status								
Normal	2194	907	17,017	17,832	1354	1344	281	178
45,X	3		3^b^		0		0	
47,XXX	0		18		0		0	
47,XXY		2		20		1		0
Total	5		41		1		0	
Total by Sex	2197	909	17,038	17,872^c^	1354	1345	281	178
Total	3106		34,910		2699		459	

WTCCC2, Wellcome Trust Case Control Consortium phase 2.

a *N* = 34,910 live-born infants from Table 1 of Nielsen and Wohlert (26) (Population Control Samples).

b This value was derived from the number of infants with 45,X syndrome (*n* = 1) and 45,X/46,XX mosaic syndrome (*n* = 2).

c 17,872 includes 20 further males with abnormal karyotypes other than 47,XXY.

**Table 2:** Frequencies of Large (>1 MB) CNVs Stratified by Type

	No. CNVs (No. Samples [%])^a^		
RDD Cases (*n* = 2723)	Screened Control Samples (*n* = 348)	WTCCC2 Control Samples (*n* = 2359)
All CNVs	77 (74 [2.71%])	4 (4 [1.10%])	45 (45 [1.91%])
Deletion CNVs	21 (20 [.73%])	2 (2 [.55%])	13 (13 [.55%])
Duplication CNVs	56 (54 [1.98%])	2 (2 [.55%])	32 (32 [1.36%])

CNV, copy number variant; RDD, recurrent depressive disorder; WTCCC2, Wellcome Trust Case Control Consortium phase 2.

a Frequencies of samples with large CNVs in our datasets are given in parentheses with percentages in brackets.

**Table 3:** Phenotype-Genotype Association Results for Tests Between Phenotype and CNVs Falling Over Regions of the Genome Previously Associated With Schizophrenia

Phenotype	CNV Type	No. Samples	*t*	*p* > |*t*|
Age of Onset	All	1926	2.12	.03^a^
	Deletions	1926	1.35	.18
	Duplications	1926	1.64	.10
Duration of Worst Episode	All	977	−.56	.58
	Deletions	977	−.14	.89
	Duplications	977	−.57	.57
Neuroticism	All	1580	−1.74	.08
	Deletions	1580	−.53	.60
	Duplications	1580	−1.71	.09
Extraversion	All	1619	1.68	.09
	Deletions	1619	1.72	.09
	Duplications	1619	.92	.36
Psychoticism	All	1619	−.21	.83
	Deletions	1619	.57	.57
	Duplications	1619	−.58	.56

CNV, copy number variant.

a *p* < .05.
