# Supplementary material for: Phenotypic Association Analyses With Copy Number Variation in Recurrent Depressive Disorder
Source: Biol Psychiatry. 2016 Feb 15;79(4):329–36. doi: 10.1016/j.biopsych.2015.02.025 (PMC4725574; doi:10.1016/j.biopsych.2015.02.025)
Supplement: Supplementary file 1 — Supplementary Material [file mmc1.pdf]

Phenotypic Association Analyses with Copy Number Variants in Recurrent Depressive Disorder

Supplementary Information

Contents

Supplemental Methods & Materials 2
Samples 2
The Depression Case Control Study 2
The Depression Network Study 3
The Genome Based Therapeutic Drugs for Depression (GENDEP) Study 3
Phenotypic Data Collection 4
Sample Quality Control 4
Derivation of a High Quality Sample Set of Rare CNVs 5
Deriving a Set of Large CNVs (>1 MB) 6
CNV Merging Definitions 6
CNV Validation 7
Supplemental Results 11
Sex Chromosome and Autosome Aneuploidies 11
Large CNVs 15
Phenotypic Analyses 21
CNVs Falling Over Regions Previously Associated With Schizophrenia 23
1q21.1 23
2p16.3 24
15q11.2 25
15q13.3 26
16p13.1 27
16p11.2 28
22q11.2 29
Whole Genome Burden Analysis 30
Supplemental References 33

Supplemental Methods & Materials

Samples

This sample is drawn from three existing cohorts of patients with recurrent unipolar depression. The mean age of sampling for our cases was 45.2 years (SD 12.2 years) with a mean age of onset of 26.1 years (SD 12.0 years). 1,692 cases (54.5%) were of non-UK European heritage. Screened control samples had a mean age of 41.4 years (SD 13.2 years) and were exclusively of UK origin. All cases were ascertained by interview with the Schedules for Clinical Assessment in Neuropsychiatry (SCAN) (1). Subjects with a history or family history of schizophrenia or bipolar disorder were excluded, as were subjects with mood symptoms secondary to alcohol or substance misuse. Subjects with mood incongruent psychotic symptoms were also excluded. 459 screened control samples (178 male and 281 female) were derived from the Bipolar Case Control study, with each potential participant screened for a past or current diagnosis of psychiatric disorder (2). A larger screened control sample was discarded from this analysis because the DNA (derived from cheek swabs) proved to be unsuitable for copy number variation (CNV) analysis. Further details of each contributing study follows.

The Depression Case Control Study

The Depression Case Control (DeCC) sample consists of 1,536 cases (69% women) of recurrent ICD-10/DSM-IV criteria diagnosed and SCAN (1; 3) assessed recurrent unipolar depressive disorder of at least moderate severity. Subjects were from three clinical UK sites in London, Cardiff and Birmingham, drawn from psychiatric clinics, hospitals and general medical practices and from volunteers responding to media advertisements. Subjects were excluded if they, or a first-degree relative, had a history of mania, hypomania, or schizophrenia. Subjects were also excluded if they experienced mood incongruent psychotic symptoms or had histories of intravenous drug use with a lifetime diagnosis of dependency or depression occurring solely consequent to substance misuse or medical problems. Mean age at recruitment was 47.5 years, SD 11.3 years, range 19-85 years. Further details on this cohort can be found in Gaysina *et al.* (4).

The Depression Network Study

1,200 probands (76% women) from an affected sib-pair linkage study called the Depression Network Study (DeNt) were included as part of this study. Individuals were recruited from psychiatric clinics, hospitals, general medical practices and from volunteers responding to media advertisements in 8 clinical sites around Europe. The probands selected were aged between 18-65 years old and consisted of ICD-10/DSM-IV criteria diagnosed and SCAN assessed cases of recurrent unipolar depressive disorder of at least moderate severity with identical phenotypic assessment to the DeCC sample. Exclusion criteria were identical to DeCC. Mean age at recruitment was 45.1 years, SD 11.8 years, range 18-78 years. All probands were of exclusively white European parentage, identified by self-report. Venous blood samples were collected from all participants. Further details on this cohort can be found in Farmer *et al.* (3).

The Genome Based Therapeutic Drugs for Depression Study

The Genome Based Therapeutic Drugs for Depression (GENDEP) study was a pharmacogenetic investigation into antidepressant response in individuals with depressive disorders. GENDEP included 868 treatment-seeking adults (63% women) diagnosed with ICD-10/DSM-IV unipolar major depression of at least moderate severity established via interview with the SCAN. Mean age at recruitment was 42.3 years, SD 11.5 years, range 19-72 years. Eligible participants were treated either with the predominantly noradrenergic drug nortriptyline or the highly selective serotonergic drug escitalopram over a 26-week period. Participants were drawn from referrals from general practice and psychiatric services and by advertisement in nine European centers. Only those with white European ethnicity were included (assessed by self-report). Potential participants were excluded if they had a family history of bipolar affective disorder or schizophrenia in a first-degree relative, a personal history of hypomanic or manic episodes, schizophrenia, mood incongruent psychotic symptoms, primary substance misuse, primary organic disease and pregnancy.

The only phenotypic difference from DeCC and DeNt is that GENDEP patients need not have suffered from recurrent disorder (although the majority, 61%, did have recurrent disorder) (5). All samples were included in this analysis. Venous blood samples were collected from all participants.

Phenotypic Data Collection

The Eysenck Personality Questionnaire (EPQ) is a three-dimensional personality taxonomy (6). The questionnaire is composed of various statements to which the subject agrees or disagrees on a five point Likert scale. The EPQ is self-administered. EPQs were either completed by subjects when they visited the center, or sent to them via post for completion at home. Scores from individual questions are aggregated into three dimensional scores of personality; neuroticism, psychoticism and extraversion, based on the EPQ manual instructions (6). In this study, the original 90-item EPQ was used. Duration of worst episode was defined as the length of time in weeks subjects could recall their worst episode of depression lasting, whilst age at onset was the subject's estimation of when they first noticed a problem with mood symptoms or behaviors.

Sample Quality Control

CNV call quality is dependent on sample quality. Artefactual calls can also arise from misalignment and physical contamination of plates during initial fluorescence intensity reading. This results in a variety of abnormal distributions of fluorescence intensity. Whilst the genotype call rate, standard deviation of the log R ratio (LRR) and the standard deviation of the B allele frequency (BAF) are broad indicators of sample quality, we have found that additional parameters are required to exclude samples with artefactual CNV calls. The LRR is a measure of the ratio of overall probe intensity compared to a canonical reference value derived from all samples at each marker. The BAF represents, in the case of biallelic markers, an allelic intensity ratio.

The waviness factor is a metric calculated by PennCNV based on the median absolute deviation of LRR values, and designed to be robust to outliers (as, for example, may occur with the occurrence of a true CNV) (7). The metric tends to be related to artefacts due to local GC nucleotide content, amongst other factors, which is not accounted for by intra and inter-sample normalization and specific correction for known artefacts caused by local GC content.

Extreme and wide LRR values are metrics designed to detect samples with excessive numbers of extreme (< -1) and wide (deviation from 0 of > 0.5) LRR values respectively. Such

samples usually have otherwise good sample quality control (QC) metrics but for reasons likely to be accountable to practical problems with microarrays during lab processing, such as window mismatching during fluorescence reading, have high numbers of LRR values that deviate from that expected assuming a diploid genotype.

Derivation of a High Quality Sample Set of Rare CNVs

A high quality sample set was created based on the following exclusion thresholds.

1. Genotype call rate < 99%
2. B allele frequency standard deviation (BAFSD) > 0.045
3. Log R ratio standard deviation (LRRSD) > 0.27
4. Waviness factor (WF) < 0.04 or > -0.04
5. Proportion of extreme LRR values (< -1) > 5,627 (> 1% of total marker number)
6. Proportion of wide LRR values (deviation from 0 > 0.5) > 28,135 (> 5% of total marker number)
7. Number of CNV calls (before QC) made by any calling method > 3SD from the mean call number for all samples

CNV calls from each algorithm were merged into a consensus call set (see information below for merging definitions).

Individual CNV calls were excluded according to the following criteria:

1. Length < 100kb
2. Calls made with < 10 consecutive markers
3. Calls where 50% of the total length of the call fell within 500 kb of the centromere or telomere of each chromosome or within regions coding for immunoglobulins
4. Calls where 20% of the total length of the call fell within regions of the genome where the analysed marker density was low (< 1 marker per 200,000 bp (*n* = 134)).

To derive a set of rare CNV calls (defined as occurring at a frequency in our sample of less than 1% of the total sample number) we used PLINK v1.07 (8) to exclude calls made over regions of the genome where the total number of calls that overlapped by at least 50% exceeded 1% of the total sample number within the analysis. PLINK was also used to calculate the total number of CNVs per sample, further stratified into deletion and duplication calls, and

to restrict our call set to those CNVs that fell over regions with previous association with schizophrenia (9) (defined as 1q21.1, 2p16.3, 15q11.2, 15q13.3, 16p13.1, 16p11.2 and 22q11.2) and to a set of singleton CNVs, again further stratified into deletion and duplication calls.

Deriving a Set of Large CNVs (>1 MB)

For the detection of large CNVs (>1 MB and >100 markers), we relaxed our QC criteria to improve power, as large CNVs are detected with confidence by CNV calling algorithms and their rarity makes visual inspection practical. We excluded samples where the LRRSD > 0.3 and the BAFSD > 0.045. All other samples were included in onward analysis, and CNV calls were derived from one algorithm (PennCNV) only. 2,723 case samples, 348 screened control samples and 2,359 WT2 control samples passed sample QC thresholds.

CNV Merging Definitions

Having processed array intensity values using three separate algorithms, we derived a call set based on the intersection of the outside boundaries of overlapping calls from the same sample made by iPattern and QuantiSNP, with PennCNV calls used for confirmation of calls, but not for boundary delineation. Hence all calls used in this analysis were restricted to those made by all three methods. Calls made as a continuous event by one method but which were broken by another method were merged into a consensus call, as illustrated in the example below

```
|----------------------------------------------------|             QuantiSNP call
     |----------|    |-----------|   |----------|   |------|        iPattern calls
|----------------------------------------------------------|       merged call
```

However if the calls were fragmented by both programs, such regions were not merged.

```
|---------------|     |---------------|    |------------------|    QuantiSNP calls
  |----------------------------|                |---------------|  iPattern calls
|-------------------------------------|    |---------------------|  merged calls
```

All CNV information is derived and reported on the basis of build 18 of the human genome reference sequence.

CNV Validation

The CNV validation sample was selected as part of a separate project using the same sample to look more closely at the 22q11.2 region, as it is of evolutionary interest. Other regions were selected on the basis of evidence that they might be pertinent CNVs in affective disorders in particular, or psychiatric disorders in general. The samples selected for the 22q11.2 region were selected randomly from a subset of samples where DNA in sufficient quantity was available for analysis. Since the selection process was not based on whether the samples had a CNV called by the Illumina array, not all samples necessarily had a called CNV that could be followed up. Samples selected from within the regions of interest other than 22q11.2 were selected on the basis that a putative CNV was available for follow up. Of 183 samples selected, 35 samples (with a total of 36 CNVs) had CNVs that could be validated given the regions covered by the array (see Tables S1 and S2).

To prepare samples, DNA concentrations and 260:280 ratios were checked before aliquoting 40 µl of DNA at 50 ng/µl into individual wells. 1 µg of sample DNA was hybridized against 1 µg of Human Genomic Male DNA (Promega, WI, USA). The samples were labelled following Agilent's aCGH protocol 6.2.1. The purified labelled samples were dried down to completeness and then hybridized onto 4x180K arrays following the manufacturer's guidelines. Arrays were washed and scanned and feature-extracted as per the manufacturer's specifications with Agilent Feature Extraction 10.7.3.1. Raw data was in the form of Log_2 ratios was normalized and displayed using Cytosure software (Oxford Genome Technologies, Oxford, UK). We then visually followed up each call using the software's plots of Log_2 ratios and compared them to the original call. X chromosome calls were not used in our analysis.

36 CNV calls were followed up, of which 36 (100%) validated. See Table S2 for details of CNVs followed up and Fig. S1 for an example of a 137 kb deletion call follow up.

Table S1. Details of CNVs (hg18 coordinates) followed up by array CGH

Sample	Chr	Start	Stop	Length	CNV Type	Validation
B007UFP	22	17,257,787	17,388,108	130,321	Dup	Validates
B007UTU	15	20,306,549	20,635,884	329,335	Del	Validates
B007UHK	6	161,239,662	161,706,684	467,022	Dup	Validates
B007UA4	10	67,920,659	68,077,867	157,208	Del	Validates
B007U6U	22	17,257,787	17,388,108	130,321	Dup	Validates
B007V4E	20	14,732,595	14,902,943	170,348	Del	Validates
B007VD5	15	20,314,760	20,778,963	464,203	Del	Validates
B007V5X	22	17,257,787	17,388,108	130,321	Del	Validates
B007V9B	22	17,257,787	17,388,108	130,321	Dup	Validates
B007VM8	15	20,314,760	20,685,685	370,925	Del	Validates
B007U64	20	14,662,457	15,030,546	368,089	Del	Validates
B007UUQ	3	7,999,019	8,253,215	254,196	Dup	Validates
B0090L4	15	20,306,549	20,778,963	472,414	Del	Validates
B007U9J	22	22,020,325	23,409,925	1,389,600	Dup	Validates
B007V7K	20	14,557,957	14,748,535	190,578	Del	Validates
B007V7K	22	17,257,787	17,388,108	130,321	Dup	Validates
B007UAC	6	162,767,020	162,903,833	136,813	Del	Validates
B007V3P	10	67,885,161	68,135,499	250,338	Del	Validates
B007V8F	22	17,257,787	17,388,108	130,321	Del	Validates
B008BPM	20	14,281,754	14,382,250	100,496	Del	Validates
B008BSK	20	14,685,843	14,788,502	102,659	Del	Validates
B008BPL	6	162,610,624	162,806,676	196,052	Del	Validates
B008BUA	6	162,824,155	162,956,501	132,346	Del	Validates
B008C0Y	20	14,691,905	14,884,935	193,030	Del	Validates
B007VNR	20	14,810,972	15,091,806	280,834	Del	Validates
B007VGG	20	14,825,654	15,000,514	174,860	Del	Validates
B007UKC	10	68,023,745	68,143,405	119,660	Del	Validates
B007UP6	6	162,719,107	162,834,976	115,869	Del	Validates
B0090V3	20	14,940,542	15,104,130	163,588	Del	Validates
B008ZU0	10	67,727,069	67,880,428	153,359	Del	Validates
B007UMW	20	14,620,177	14,915,506	295,329	Del	Validates
B008CBL	22	17,257,787	17,388,108	130,321	Del	Validates
B008BXP	22	17,257,787	17,388,108	130,321	Del	Validates
B008C5F	6	162,637,688	162,834,976	197,288	Dup	Validates
B008CBC	6	162,636,531	162,829,925	193,394	Dup	Validates
B008C30	20	14,300,394	14,414,618	114,224	Del	Validates

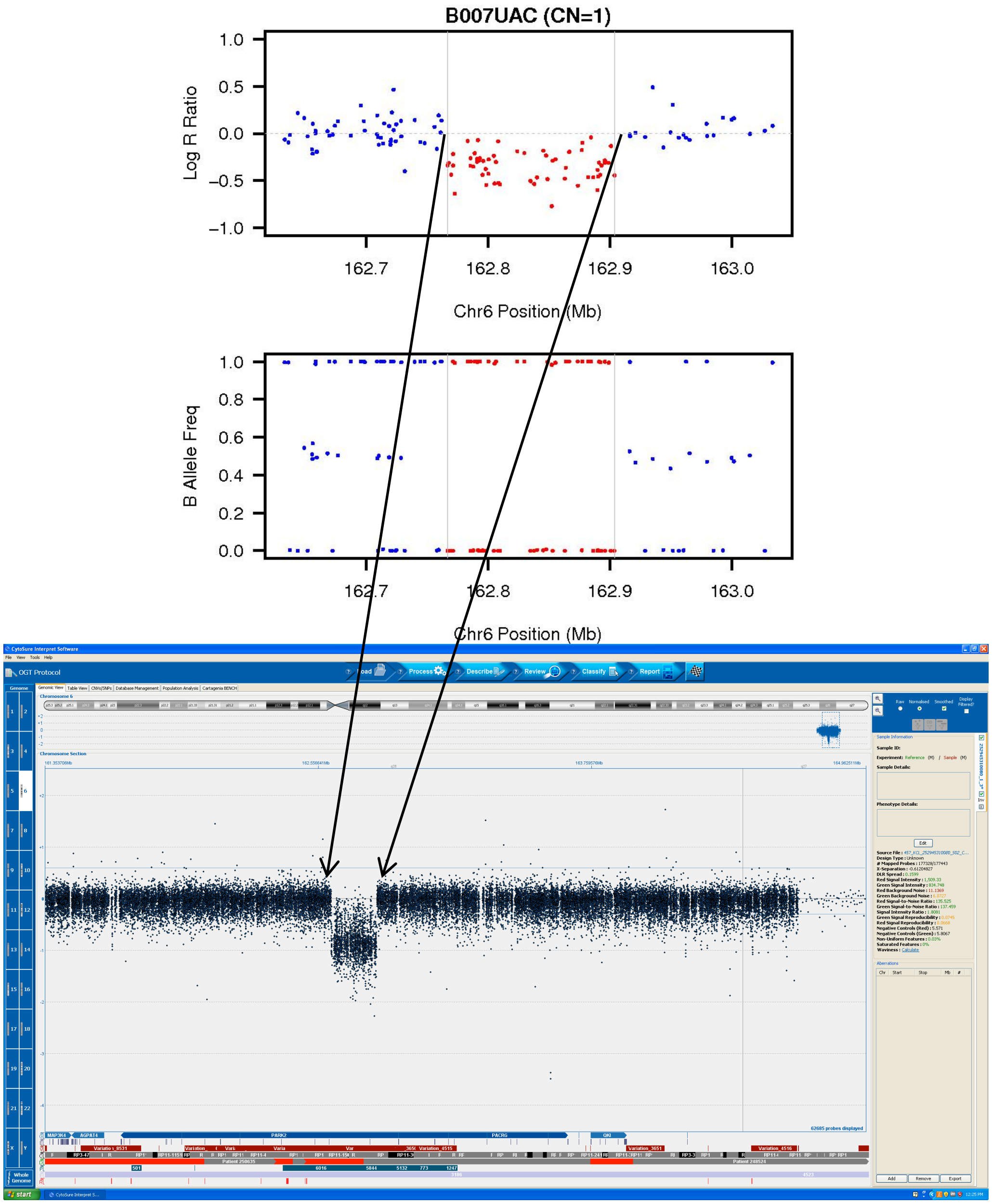

Figure S1. A 137 kb deletion CNV call in the *PARK2* gene is validated with high density array CGH.

Table S2. Genomic regions covered by CGH array follow up of rare CNVs. X chromosome calls were not used in our analysis.

chr3:5,997,636-8,638,508
chr3:53,389,717-55,517,902
chr6:160,833,017-164,666,324
chr10:59,301,152-60,900,609
chr10:67,334,811-69,148,660
chr12:1,870,350-2,688,889
chr15:20,195,144-20,872,219
chr20:13,859,307-16,039,340
chr22:16,300,001-24,300,000
chrX: 5,390,291-8,806,740
Total coverage: 30,524,214bp

Supplemental Results

Sex Chromosome and Autosome Aneuploidies

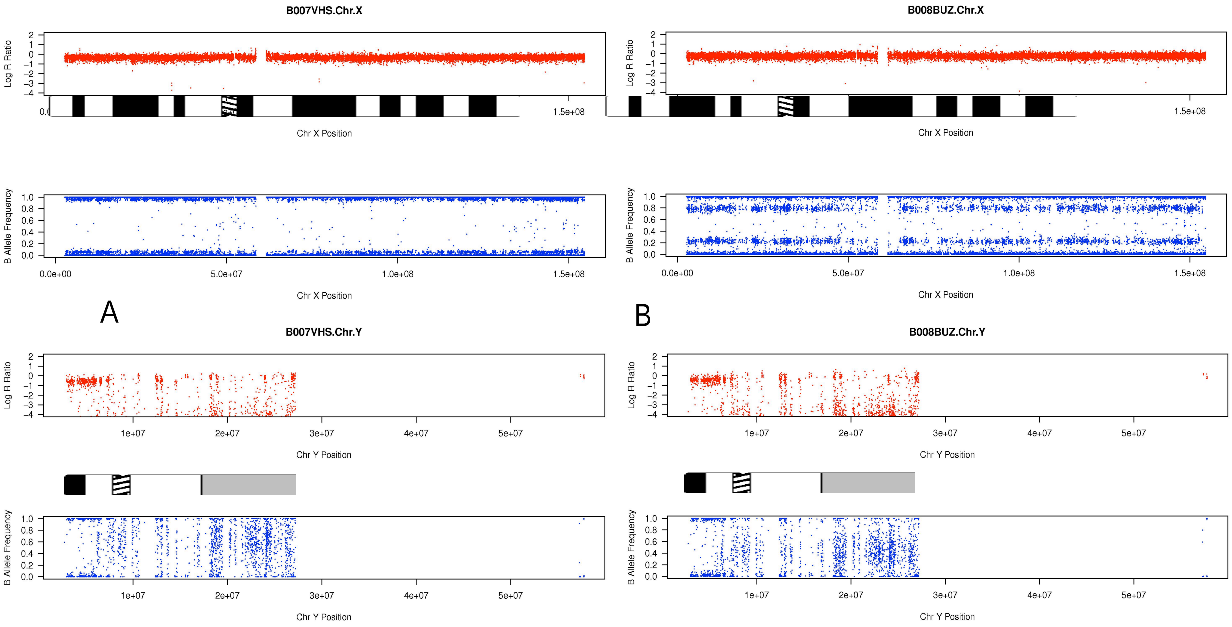

Figure S2. Two cases of Turner's syndrome in recurrent depressive disorder cases. (**A**) Phenotypic female with evidence of 1 X chromosome (log R ratio is shifted below 0 for most markers and B allele frequency plot indicates complete homozygosity) and no Y chromosome (Y chromosome markers are randomly distributed). (**B**) Phenotypic female with evidence of X/XX mosaicism (split B allele frequency plot) and no Y chromosome.

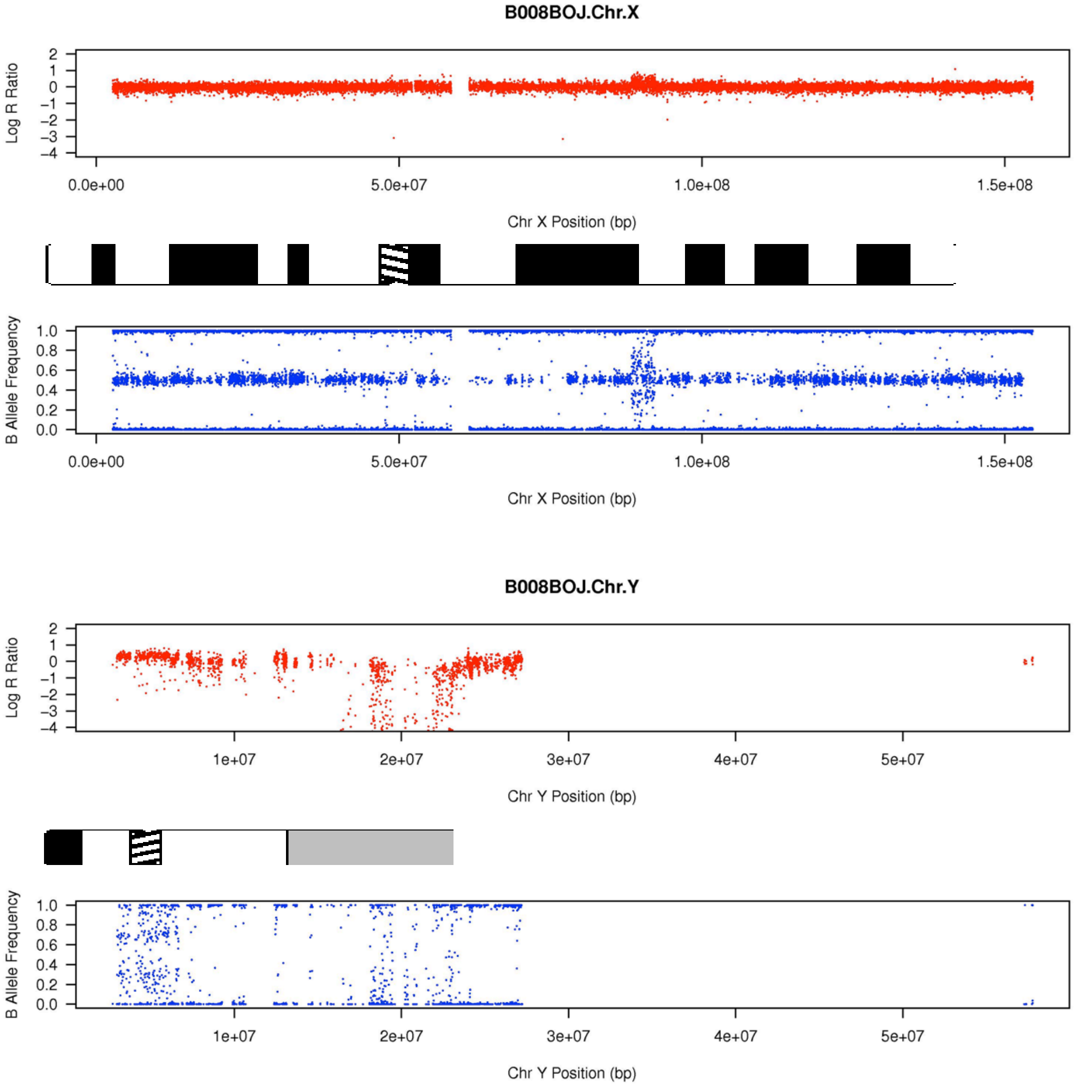

Figure S3. Klinefelter's syndrome with an additional deletion of Yq. Phenotypic male with evidence of two X chromosomes and a Y chromosome with a deletion of Yq.

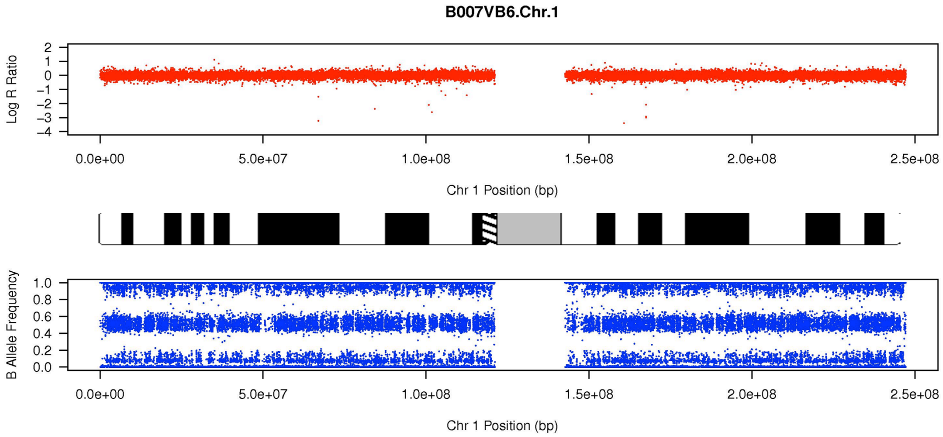

Figure S4. 10-20% diploid/triploid mosaicism. The B allele frequency plot shows a five-way split indicative of a triploid cell line with genotypes not present in the euploid cells (plot for chromosome 1 only shown).

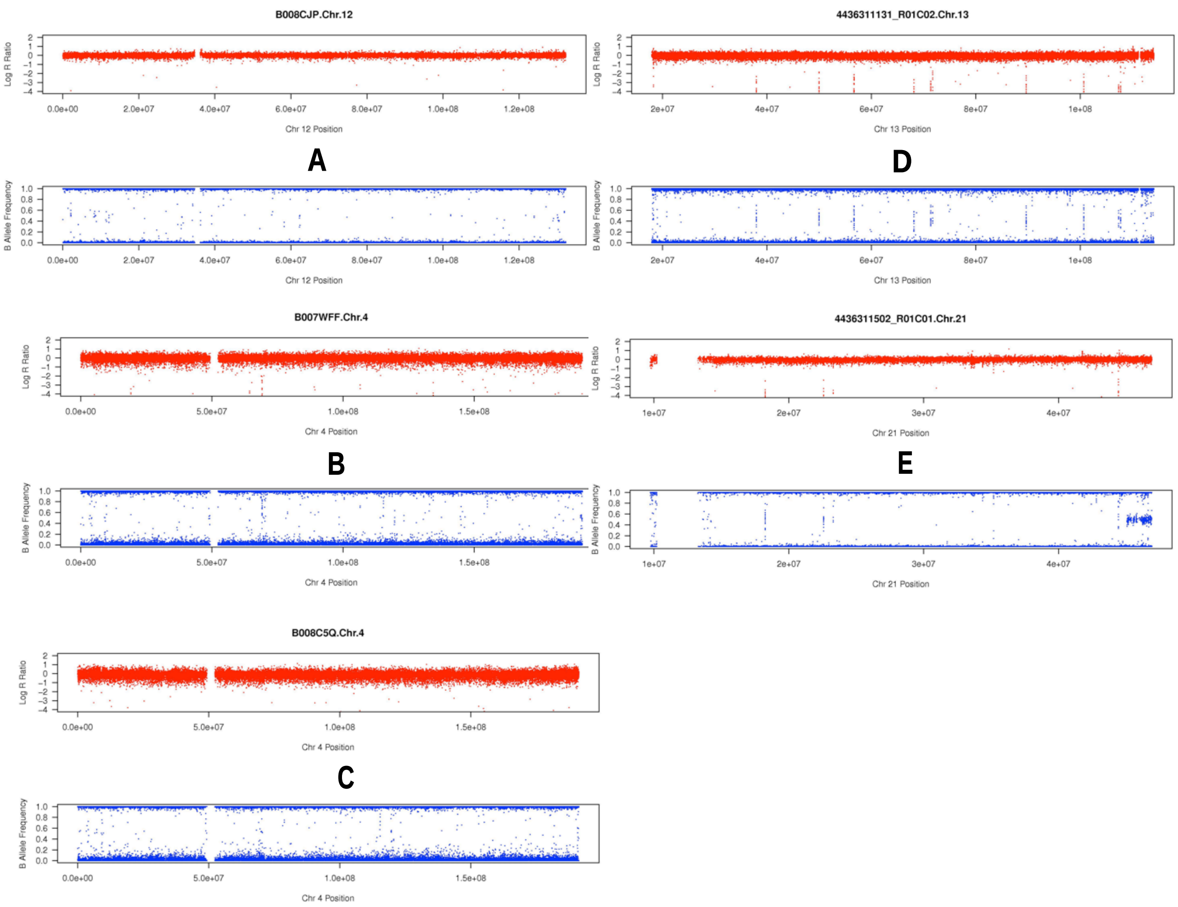

Figure S5. Five examples of uniparental isodisomy with meiotic origin. Compare plots of the B allele frequency (blue), indicating homozygosity, to those seen in other figures in this document, and far right of figure e (incomplete isodisomy). Figs. a, b & c (left column). 3 cases. 1 case with complete isodisomy for chromosome 12, and 2 cases with complete isodisomy for chromosome 4. Figs. d & e (right column). 2 WT2 controls, 1 with complete isodisomy for chromosome 13 and 1 with almost complete isodisomy for chromosome 21, suggesting an origin during or after recombination in meiosis II.

Large CNVs

Details of all large CNVs >1 MB are given in Table S3. The largest variant was a 9.16 MB heterozygous deletion of chromosome 13 in a case (Fig. S6A). Two further large duplication CNVs (8.46 MB and 8.45 MB) were observed in the unscreened WT2 sample, on chromosomes 6 and 11 respectively (Fig. S6B & Fig. S6C). The next largest variant was a 4.5 MB heterozygous duplication of chromosome 10 in a case sample (Fig. S6D). The largest CNV in a case to exhibit psychotic symptoms was a 2.2 MB duplication CNV in chromosome 18 (Fig. S6E). One case sample (without psychotic symptoms) carried the 22q11.2 microdeletion (Fig. S6F).

Table S3. Table of all called CNVs >1 MB. Human Genome build hg18 coordinates.

Chr	Start	End	Length (bp)	Marker no.	CN	Sample ID	Cohort
1	73386148	74566707	1,180,559	157	1	B008BOC	Case
1	104256030	106108011	1,851,981	360	1	B007V0G	Case
1	144967972	146325557	1,357,585	288	3	B007UO6	Case
2	11060172	12577917	1,517,745	401	1	B007VCD	Case
2	106501602	110340339	3,838,737	628	1	B007U6Z	Case
2	106501602	107807545	1,305,943	281	1	B007UKB	Case
3	1008035	2021410	1,013,375	482	3	B007UTC	Case
3	59591557	60835908	1,244,351	629	3	B007V5Z	Case
3	95067163	96646248	1,579,085	186	3	B007V1A	Case
3	105373669	107260259	1,886,590	484	3	B007VE5	Case
3	108791667	111483418	2,691,751	510	3	B007UN0	Case
3	144675123	147281477	2,606,354	533	3	B008CJK	Case
4	31349107	32829156	1,480,049	230	1	B007VGF	Case
4	35064787	37341072	2,276,285	505	1	B007UJM	Case
4	69700559	70753827	1,053,268	297	3	B007VNI	Case
4	171384920	173222335	1,837,415	276	1	B007V3M	Case
4	182348352	184232583	1,884,231	655	3	B007VAZ	Case
5	22507055	23665834	1,158,779	230	3	B008BZR	Case
5	68950807	70702961	1,752,154	285	3	B0090BA	Case
5	68950807	70672298	1,721,491	282	3	B007WED	Case
5	69096757	70523956	1,427,199	242	3	B0090SJ	Case
5	69247965	70672298	1,424,333	243	3	B007UZE	Case
5	103794893	104873156	1,078,263	224	3	B0090RX	Case
6	161001321	162157939	1,156,618	395	3	B0090AE	Case
6	161609806	162809965	1,200,159	474	3	B007UG6	Case
6	162097013	163409658	1,312,645	414	1	B008CC2	Case
6	162394883	164603705	2,208,822	704	3	B0090AE	Case
6	162724494	163943698	1,219,204	386	1	B007V0A	Case
6	165662355	167031058	1,368,703	512	3	B0090AE	Case
7	14327131	15466197	1,139,066	340	3	B007V35	Case
7	14542688	15792716	1,250,028	388	3	B007VFE	Case
7	61256909	62336389	1,079,480	104	3	B008BZL	Case
7	87986401	89347872	1,361,471	306	3	B007V4Q	Case
7	88186382	89722103	1,535,721	361	3	B008CE4	Case
7	117177208	118948979	1,771,771	242	3	B008C6W	Case
8	2324501	5187543	2,863,042	1919	3	B007U70	Case
8	5438789	6913279	1,474,490	759	1	B007VZK	Case
8	35290651	36666471	1,375,820	116	3	B008C10	Case
8	99482435	101154954	1,672,519	143	3	B007UUX	Case
9	10639385	11786468	1,147,083	260	1	B007UKF	Case
10	46021623	47211888	1,190,265	149	3	B008BTE	Case
10	46111616	47218918	1,107,302	136	3	B0090AI	Case
10	46132717	47218918	1,086,201	135	3	B008ZUK	Case
10	46132717	47218918	1,086,201	135	3	B007UNW	Case
10	46132717	47218918	1,086,201	135	3	B007UFJ	Case
10	46132717	47211888	1,079,171	134	3	B008C5C	Case
10	46158713	47218918	1,060,205	131	3	B008BQW	Case
10	46213921	47218918	1,004,997	124	3	B008ZV6	Case
10	65131646	66391943	1,260,297	224	3	B0090U9	Case

Chr	Start	End	Length (bp)	Marker no.	CN	Sample ID	Cohort
10	84022134	88561319	4,539,185	915	3	B008BO1	Case
10	129492808	131123023	1,630,215	517	3	B007W9T	Case
11	103488072	106417004	2,928,932	506	1	B007VEM	Case
12	83410704	85288749	1,878,045	269	1	B007WFG	Case
13	22235187	24201255	1,966,068	669	3	B008BVQ	Case
13	66653275	75813311	9,160,036	2214	1	B007UJM	Case
13	91325180	92925678	1,600,498	384	3	B007U93	Case
15	18530950	20049770	1,518,820	146	3	B007ULT	Case
15	18752062	20093116	1,341,054	129	3	B007UYX	Case
15	18752062	20093116	1,341,054	129	3	B007U5X	Case
15	18788683	20093116	1,304,433	127	3	B007UTV	Case
15	18822301	20016954	1,194,653	120	3	B007V0F	Case
15	19095051	20306549	1,211,498	109	3	B00909T	Case
15	26717846	28169412	1,451,566	320	3	B007V4K	Case
15	26717846	28124669	1,406,823	311	3	B008BW9	Case
15	27000239	28156445	1,156,206	303	1	B008BO7	Case
15	27081472	28156445	1,074,973	285	1	B00908J	Case
16	12109192	13492401	1,383,209	447	1	B008ZTQ	Case
16	16767302	18075924	1,308,622	330	3	B007UMC	Case
17	14030694	15416569	1,385,875	482	1	B008CHY	Case
17	14030694	15399033	1,368,339	479	1	B007UFP	Case
18	36397862	38557752	2,159,890	449	3	B007V4B	Case
21	13484385	16409990	2,925,605	636	3	B007UVL	Case
22	17202486	19792353	2,589,867	518	1	B007ULM	Case
22	17241748	18686993	1,445,245	377	3	B008C2Z	Case
22	17241748	18621160	1,379,412	364	3	B0090SV	Case
22	22013796	23327473	1,313,677	317	3	B007WGC	Case
22	22038020	23327473	1,289,453	307	3	B0090TI	Case
1	197301529	198317897	1,016,368	202	3	B0095FA	Scr Control
2	106479306	107807545	1,328,239	284	1	B0095HF	Scr Control
10	66813888	67895016	1,081,128	316	1	B0095AQ	Scr Control
11	48675981	50478883	1,802,902	265	3	B00958N	Scr Control
1	144943150	146293282	1,350,132	232	1	4439467553_R01C01	WT2
1	144943150	146293282	1,350,132	232	1	4436303623_R01C02	WT2
1	144943150	146293282	1,350,132	232	3	4436320275_R01C01	WT2
1	144967972	146293282	1,325,310	231	1	4435415116_R01C02	WT2
2	63430545	64601060	1,170,515	171	3	4436320620_R01C02	WT2
2	106245033	107807545	1,562,512	278	1	4436311428_R01C01	WT2
3	1118424	2183832	1,065,408	434	3	4435415367_R01C01	WT2
3	1179591	2322522	1,142,931	466	1	4436303459_R01C01	WT2
3	59684090	60998395	1,314,305	630	3	4436311391_R01C01	WT2
3	113464022	114495199	1,031,177	245	1	4436320538_R01C02	WT2
4	59890867	61454026	1,563,159	221	3	4436311783_R01C02	WT2
4	87295384	88321991	1,026,607	144	3	4436303306_R01C01	WT2
4	116138572	117727147	1,588,575	181	1	4436320061_R01C02	WT2
4	188356006	190029985	1,673,979	344	3	4435415073_R01C02	WT2
4	188704457	190189143	1,484,686	316	3	4436311045_R01C02	WT2
5	103816450	104873156	1,056,706	168	1	4436311900_R01C02	WT2
6	64788974	73250711	8,461,737	1656	3	4435415150_R01C01	WT2
6	65228140	66370871	1,142,731	196	1	4436311565_R01C02	WT2
6	86782732	87884847	1,102,115	173	3	4435415347_R01C02	WT2

**Chr**	**Start**	**End**	**Length (bp)**	**Marker no.**	**CN**	**Sample ID**	**Cohort**
7	9700848	10792719	1,091,871	240	1	4436320770_R01C02	WT2
7	10242504	11763426	1,520,922	455	3	4436311699_R01C01	WT2
7	88475318	89718885	1,243,567	266	3	4436303704_R01C01	WT2
7	109614824	111159480	1,544,656	253	3	4436311172_R01C02	WT2
8	12993322	14169180	1,175,858	467	1	4435415680_R01C02	WT2
11	739776	9194116	8,454,340	1976	3	4436303648_R01C02	WT2
11	36552176	37717260	1,165,084	208	3	4436320284_R01C01	WT2
13	22425297	23860983	1,435,686	506	3	4436311021_R01C02	WT2
13	22425297	23846927	1,421,630	501	3	4436311747_R01C02	WT2
13	22437563	23575307	1,137,744	406	3	4436320643_R01C02	WT2
13	22437563	23559833	1,122,270	394	3	4439467575_R01C02	WT2
13	55696577	56711568	1,014,991	101	1	4435415621_R01C02	WT2
14	42938662	44466915	1,528,253	266	1	4692770059_R01C02	WT2
15	28723577	30302218	1,578,641	290	3	4436320023_R01C02	WT2
15	30713368	32587887	1,874,519	693	3	4436320692_R01C01	WT2
16	15032942	16190572	1,157,630	267	3	4436320741_R01C01	WT2
16	15032942	16076966	1,044,024	184	3	4687760022_R01C01	WT2
16	15032942	16076109	1,043,167	183	3	4435415225_R01C01	WT2
17	31889664	33297438	1,407,774	259	3	4440783092_R01C02	WT2
21	19470131	20581408	1,111,277	235	3	4436311689_R01C02	WT2
22	17257787	19792353	2,534,566	521	3	4439467321_R01C01	WT2
22	17257787	19792353	2,534,566	521	3	4436320121_R01C01	WT2
22	17257787	18686993	1,429,206	347	3	4436320075_R01C02	WT2
22	17718412	19792353	2,073,941	440	3	4429373008_R01C01	WT2
22	21328337	23326630	1,998,293	367	3	4436303748_R01C01	WT2
22	41000055	42210450	1,210,395	329	3	4436311376_R01C01	WT2

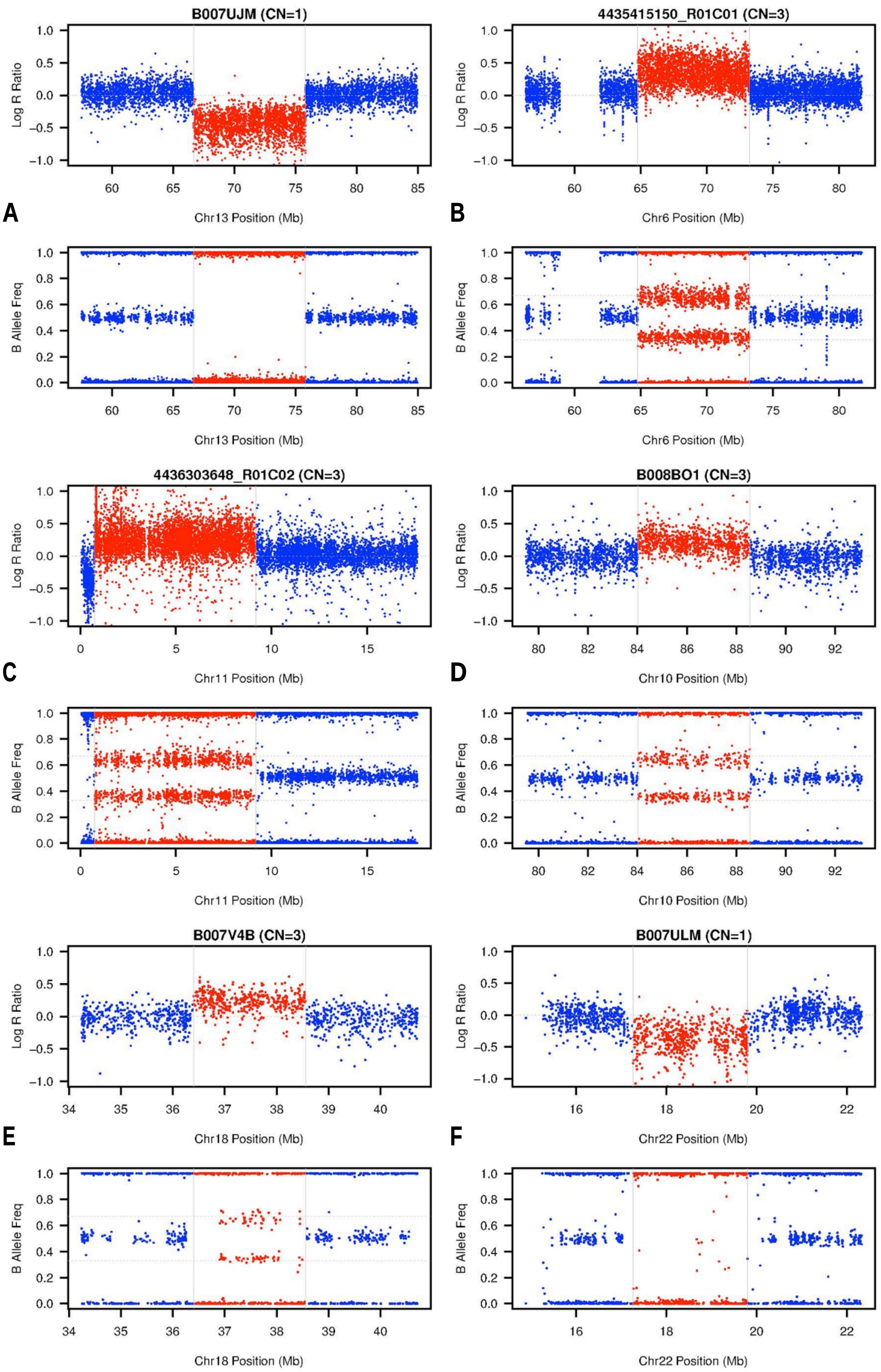

Figure S6. Six examples of large CNVs in this dataset. (**A**) 9.16 MB deletion of chromosome 13 in a case. (**B**) 8.46 MB duplication in chromosome 6 in an unscreened control sample. (**C**) 8.45 MB

duplication in chromosome 11 in an unscreened control sample. (**D**) 4.54 MB duplication in chromosome 10 in a case sample. (**E**) 2.16 MB duplication in a case with psychotic symptoms. (**F**) 2.59 MB deletion over the velocardiofacial syndrome region of chromosome 22q11.2 in a case sample without psychotic symptoms.

Phenotypic Analyses

Table S4. Phenotype-genotype association results for tests between phenotype and global rare CNV burden.

Phenotype	CNV Type	No. Samples	*t*	*P* >|*t*|	Coefficient	SE
Age of Onset	All	1,926	1.39	0.17	0.030	0.009
	Deletions	1,926	-0.03	0.97	-0.001	0.000
	Duplications	1,926	1.67	0.10	0.036	0.014
Duration of Worst Episode	All	977	0.46	0.64	0.014	0.008
	Deletions	977	0.43	0.67	0.013	0.010
	Duplications	977	0.08	0.94	0.002	0.002
Neuroticism	All	1,580	0.87	0.38	0.021	0.115
	Deletions	1,580	1.30	0.20	0.032	0.240
	Duplications	1,580	-0.08	0.93	-0.002	-0.014
Extraversion	All	1,619	0.43	0.67	0.011	0.068
	Deletions	1,619	0.29	0.77	0.007	0.066
	Duplications	1,619	0.27	0.79	0.007	0.057
Psychoticism	All	1,619	0.54	0.59	0.013	0.012
	Deletions	1,619	0.95	0.34	0.023	0.030
	Duplications	1,619	-0.28	0.78	-0.007	-0.008

Table S5. Phenotype-genotype association results for tests between phenotype and rare singleton CNVs.

Phenotype	CNV Type	No. Samples	*t*	*P* >|*t*|	Coefficient	SE
Age of Onset	All	1,926	0.79	0.43	0.0169	0.0081
	Deletions	1,926	0.42	0.67	0.0091	0.0067
	Duplications	1,926	0.66	0.51	0.0142	0.0088
Duration of Worst Episode	All	977	0.28	0.78	0.0083	0.0075
	Deletions	977	0.07	0.95	0.0020	0.0027
	Duplications	977	0.31	0.76	0.0094	0.0113
Neuroticism	All	1,580	0.07	0.95	0.0017	0.0151
	Deletions	1,580	-0.20	0.84	-0.0049	-0.0690
	Duplications	1,580	0.25	0.80	0.0062	0.0713
Extraversion	All	1,619	0.15	0.88	0.0038	0.0402
	Deletions	1,619	0.35	0.72	0.0089	0.1458
	Duplications	1,619	-0.10	0.92	-0.0025	-0.0341
Psychoticism	All	1,619	0.89	0.37	0.0219	0.0338
	Deletions	1,619	0.09	0.93	0.0022	0.0053
	Duplications	1,619	1.06	0.29	0.0260	0.0508

CNVs Falling Over Regions Previously Associated With Schizophrenia

1q21.1

Table S6. Phenotype-genotype association results for tests between phenotype and CNVs falling over 1q21.1.

CNV Region	Phenotype	No. Samples	No. CNVs	*t*	*P*>|*t*|	Coefficient	SE
1q21.1	Age of Onset	1,926	5	-0.21	0.83	-0.022	0.102
	Duration of Worst Episode	977	2	1.41	0.16	0.435	0.308
	Neuroticism	1,580	5	-0.50	0.62	-0.989	1.985
	Extraversion	1,619	5	-0.72	0.47	-1.794	2.481
	Psychoticism	1,619	5	0.37	0.71	0.125	0.342

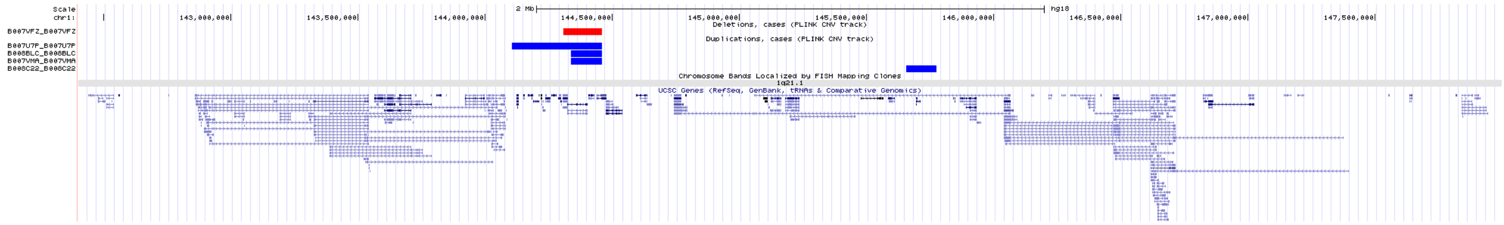

Figure S7. CNVs falling over region 1q21.1. Deletions appear in red, duplications in blue.

2p16.3

Table S7. Phenotype-genotype association results for tests between phenotype and CNVs falling over 2p16.3.

CNV Region	Phenotype	No. Samples	No. CNVs	*t*	*P* >|*t*|	Coefficient	SE
2p16.3	Age of Onset	1,926	4	0.12	0.90	0.014	0.114
	Duration of Worst Episode	977	2	-1.08	0.28	-0.334	0.309
	Neuroticism	1,580	3	-0.54	0.59	-1.379	2.559
	Extraversion	1,619	3	1.37	0.17	4.378	3.198
	Psychoticism	1,619	3	0.35	0.73	0.153	0.441

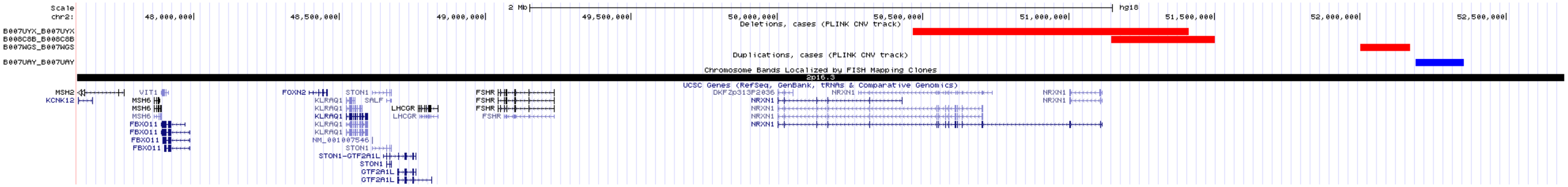

Figure S8. CNVs falling over region 2p16.3. Deletions appear in red, duplications in blue.

15q11.2

Table S8. Phenotype-genotype association results for tests between phenotype and CNVs falling over 15q11.2.

CNV Region	Phenotype	No. Samples	No. CNVs	*t*	*P* >|*t*|	Coefficient	SE
15q11.2	Age of Onset	1,926	42	1.25	0.21	0.045	0.036
	Duration of Worst Episode	977	15	-0.29	0.77	-0.034	0.114
	Neuroticism	1,580	34	-1.42	0.16	-1.143	0.805
	Extraversion	1,619	34	1.37	0.17	1.318	0.962
	Psychoticism	1,619	34	-0.40	0.69	-0.053	0.133

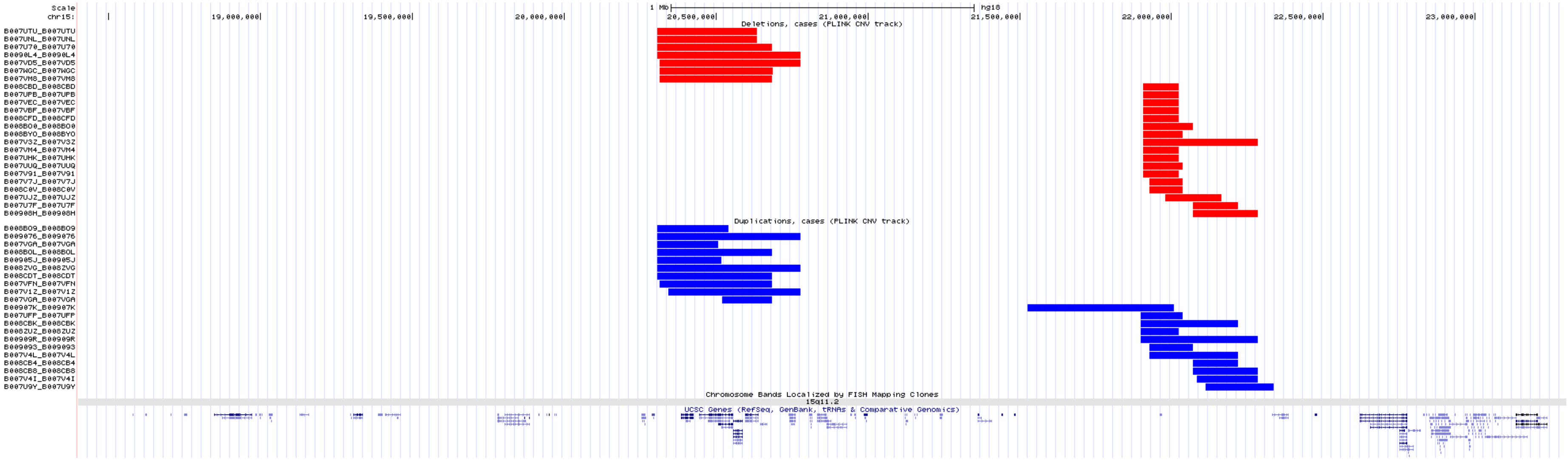

Figure S9. CNVs falling over region 15q11.2. Deletions appear in red, duplications in blue.

15q13.3

Table S9. Phenotype-genotype association results for tests between phenotype and CNVs falling over 15q13.3.

CNV Region	Phenotype	No. Samples	No. CNVs	*t*	*P* >|*t*|	Coefficient	SE
15q13.3	Age of Onset	1,926	10	1.90	0.06	0.138	0.072
	Duration of Worst Episode	977	3	-0.45	0.65	-0.113	0.252
	Neuroticism	1,580	10	-1.04	0.30	-1.457	1.406
	Extraversion	1,619	10	1.10	0.27	1.931	1.757
	Psychoticism	1,619	10	0.74	0.46	0.180	0.242

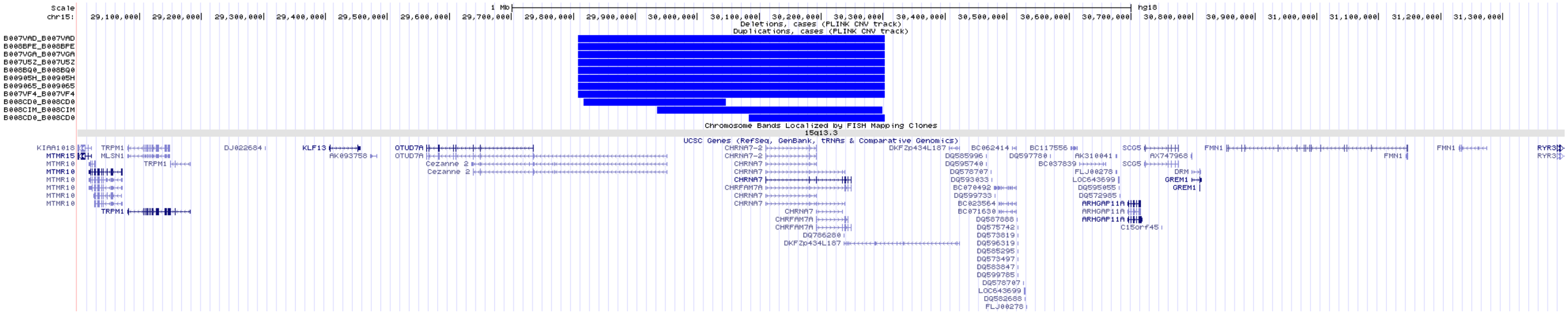

Figure S10. CNVs falling over region 15q13.3. Deletions appear in red, duplications in blue.

16p13.1

Table S10. Phenotype-genotype association results for tests between phenotype and CNVs falling over 16p13.1.

CNV Region	Phenotype	No. Samples	No. CNVs	*t*	*P* >|*t*|	Coefficient	SE
16p13.1	Age of Onset	1,926	6	-0.66	0.51	-0.062	0.093
	Duration of Worst Episode	977	1	0.85	0.40	0.371	0.437
	Neuroticism	1,580	5	-0.41	0.68	-0.820	1.986
	Extraversion	1,619	5	1.63	0.10	4.043	2.480
	Psychoticism	1,619	5	0.09	0.93	0.031	0.342

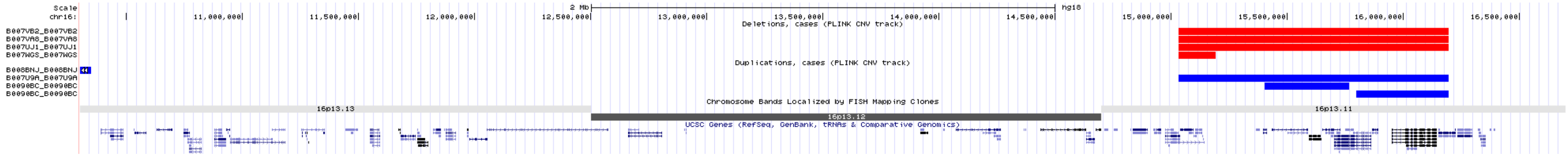

Figure S11. CNVs falling over region 16p13.1. Deletions appear in red, duplications in blue.

16p11.2

Table s11. Phenotype-genotype association results for tests between phenotype and CNVs falling over 16p11.2.

CNV Region	Phenotype	No. Samples	No. CNVs	*t*	*P* >|*t*|	Coefficient	SE
16p11.2	Age of Onset	1,926	1	0.35	0.73	0.080	0.229
	Duration of Worst Episode	977	1	-1.55	0.12	-0.674	0.436
	Neuroticism	1,580	0	N/A*	N/A*	N/A*	N/A*
	Extraversion	1,619	0	N/A*	N/A*	N/A*	N/A*
	Psychoticism	1,619	0	N/A*	N/A*	N/A*	N/A*

*No individuals with CNVs in 16p11.2 and personality trait data were available for analysis.

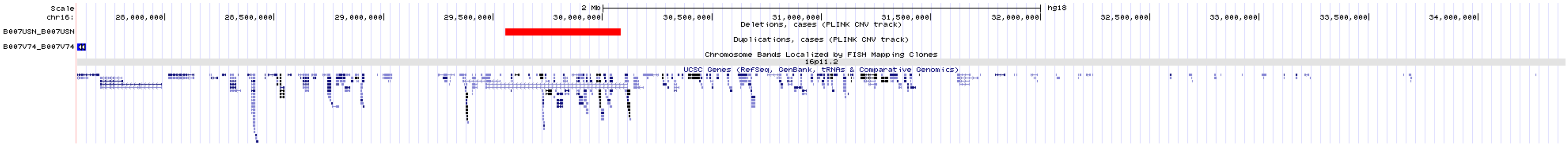

Figure S12. CNVs falling over region 16p11.2. Deletions appear in red, duplications in blue.

22q11.2

Table S12. Phenotype-genotype association results for tests between phenotype and CNVs falling over 22q11.2.

CNV Region	Phenotype	No. Samples	No. CNVs	*t*	*P* >|*t*|	Coefficient	SE
22q11.2	Age of Onset	1,926	33	1.33	0.18	0.054	0.041
	Duration of Worst Episode	977	15	-0.64	0.52	-0.072	0.113
	Neuroticism	1,580	28	-0.56	0.57	-0.483	0.861
	Extraversion	1,619	28	-0.18	0.86	-0.187	1.056
	Psychoticism	1,619	28	-0.15	0.88	-0.021	0.146

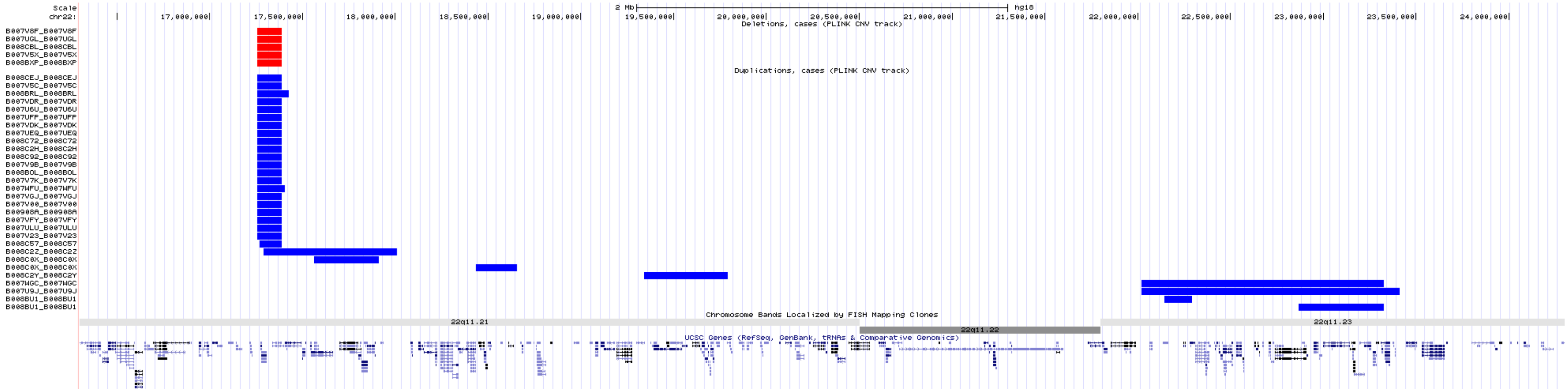

Figure S13. CNVs falling over region 22q11.2. Deletions appear in red, duplications in blue.

Whole Genome Burden Analysis

The burden of CNVs refers to the number and size of CNVs seen in individual samples. Burden analyses compare aggregates of individual measures in different cohorts. Whole genome burden analysis was performed using rare CNVs above 100 kb in size, stratified into all CNVs, deletion CNVs and duplication CNVs, and further stratified into those covering genic regions. 2,397 cases, 332 screened controls and 2,151 WT2 controls passed sample QC. Analysis for statistical significance was performed using PLINK v1.07. Results are shown for 1 and 2 sided tests using 10,000 null permutations of case-control status. Six tests performed within PLINK were performed and defined as follows:

1. RATE- The number of CNVs per sample
2. PROP- The proportion of samples with at least 1 CNV
3. KBTOT- The total length of CNVs in kilobases.
4. KBAVG- The average length of CNVs in kilobases.
5. GRATE- Number of genes spanned by CNVs.
6. GPROP- Number of CNVs with at least one gene.
7. GRICH- Number of genes per kilobase of CNV.

Full results are shown in Table S13.

The results show that there is no statistically significant evidence for an increased rate of deletion CNVs in cases when compared to controls (Rate = 0.32 vs. 0.31 respectively, 2 sided $p = 0.26$ & Prop = 0.27 vs 0.26 respectively, 2 sided $p = 0.55$) (in contrast to our previous result), however of those samples that do have a rare deletion CNV, it is on average significantly larger in cases than controls (Kbtot = 340 kb vs. 280 kb, 2 sided $p = 0.011$), and tends to cover more genes in cases than controls (Grate = 0.78 vs 0.32, 2 sided $p = 0.0002$) (in agreement with our previous result). Within this analysis duplications appear more frequently in the WT2 control group than cases, a non-significant trend for which is also seen when screened controls are compared to cases. The results highlight the significant variation that can occur when calling CNVs using different methodologies.

Table S13. Whole genome CNV burden analyses. *P* values < 0.05 are highlighted in bold. *P* values < 0.00079 (Bonferroni corrected) are highlighted in red.

Cohorts	CNV type	Test	1 sided emp. *p* value	2 sided emp. *p* value	Cases	Controls
Cases vs. all controls	All	RATE	0.91	0.21	0.69	0.72
		PROP	0.66	0.72	0.49	0.5
		KBTOT	0.36	0.7	460	450
		KBAVG	0.43	0.86	310	310
		GRATE	**0.036**	0.07	1.5	1.2
		GPROP	0.94	0.13	0.31	0.33
		GRICH	0.27	0.55	0.006	0.0058
	Del	RATE	0.14	0.26	0.32	0.31
		PROP	0.29	0.55	0.27	0.26
		KBTOT	**0.0078**	**0.011**	340	280
		KBAVG	0.068	0.13	270	240
		GRATE	**0.0002**	**0.0002**	0.78	0.32
		GPROP	0.46	0.9	0.13	0.13
		GRICH	**0.046**	0.092	0.0049	0.0041
	Dup	RATE	0.99	**0.012**	0.36	0.41
		PROP	0.98	**0.049**	0.29	0.32
		KBTOT	0.69	0.62	450	470
		KBAVG	0.53	0.93	360	360
		GRATE	0.97	0.054	0.74	0.91
		GPROP	0.99	**0.02**	0.2	0.23
		GRICH	0.79	0.43	0.0068	0.0072
Cases vs. screened controls	All	RATE	0.88	0.26	0.69	0.74
		PROP	0.75	0.56	0.49	0.51
		KBTOT	0.26	0.5	460	420
		KBAVG	0.3	0.56	310	300
		GRATE	**0.025**	0.097	1.5	0.92
		GPROP	0.77	0.54	0.31	0.33
		GRICH	0.12	0.25	0.006	0.0052
	Del	RATE	0.43	0.85	0.32	0.32
		PROP	0.58	0.9	0.27	0.27
		KBTOT	0.062	0.19	340	260
		KBAVG	0.096	0.23	270	220
		GRATE	**0.0096**	0.072	0.78	0.21
		GPROP	0.35	0.66	0.13	0.12
		GRICH	**0.041**	0.097	0.0049	0.0033
	Dup	RATE	0.96	0.084	0.36	0.43
		PROP	0.97	0.089	0.29	0.34
		KBTOT	0.3	0.57	450	430
		KBAVG	0.33	0.62	360	340
		GRATE	0.45	0.85	0.74	0.71
		GPROP	0.94	0.15	0.2	0.23
		GRICH	0.5	0.98	0.0068	0.0068

Cohorts	CNV type	Test	1 sided emp. *p* value	2 sided emp. *p* value	Cases	Controls
**Cases vs. WT2 controls**	**All**	RATE	0.86	0.29	0.69	0.71
		PROP	0.6	0.83	0.49	0.49
		KBTOT	0.43	0.84	460	450
		KBAVG	0.49	0.97	310	310
		GRATE	0.083	0.16	1.5	1.3
		GPROP	0.93	0.14	0.31	0.33
		GRICH	0.39	0.78	0.006	0.0059
	**Del**	RATE	0.13	0.25	0.32	0.3
		PROP	0.25	0.48	0.27	0.26
		KBTOT	**0.016**	**0.034**	340	280
		KBAVG	0.11	0.22	270	240
		GRATE	**0.0002**	**0.0006**	0.78	0.33
		GPROP	0.52	1	0.13	0.13
		GRICH	0.093	0.19	0.0049	0.0043
	**Dup**	RATE	0.99	**0.02**	0.36	0.41
		PROP	0.96	0.09	0.29	0.31
		KBTOT	0.76	0.48	450	470
		KBAVG	0.59	0.81	360	360
		GRATE	0.98	**0.033**	0.74	0.94
		GPROP	0.99	**0.031**	0.2	0.23
		GRICH	0.82	0.38	0.0068	0.0073

Supplemental References

1. Wing J, Babor T, Brugha T, Burke J, Cooper J, Giel R, *et al.* (1990): SCAN. Schedules for Clinical Assessment in Neuropsychiatry. *Archives of General Psychiatry* 47: 589–593.

2. Gaysina D, Cohen-Woods S, Chow P, Martucci L, Schosser A, Ball H, *et al.* (2009): Association of the dystrobrevin binding protein 1 gene (DTNBP1) in a bipolar case-control study (BACCS). *Am J Med Genet B Neuropsychiatr Genet* 150B: 836–844.

3. Farmer A, Breen G, Brewster S, Craddock N, Gill M, Korszun A, *et al.* (2004): The Depression Network (DeNT) Study: methodology and sociodemographic characteristics of the first 470 affected sibling pairs from a large multi-site linkage genetic study. *BMC Psychiatry* 4: 42.

4. Gaysina D, Cohen S, Craddock N, Farmer A, Hoda F, Korszun A, *et al.* (2008): No association with the 5,10-methylenetetrahydrofolate reductase gene and major depressive disorder: results of the depression case control (DeCC) study and a meta-analysis. *Am J Med Genet B Neuropsychiatr Genet* 147B: 699–706.

5. Uher R, Maier W, Hauser J, Marusic A, Schmael C, Mors O, *et al.* (2009): Differential efficacy of escitalopram and nortriptyline on dimensional measures of depression. *Br J Psychiatry* 194: 252–259.

6. Eysenck H, Eysenck S (1964): *Manual of the Eysenck Personality Questionnaire*. 1st ed. London: Hodder and Stoughton.

7. Diskin S, Li M, Hou C, Yang S, Glessner J, Hakonarson H, *et al.* (2008): Adjustment of genomic waves in signal intensities from whole-genome SNP genotyping platforms. *Nucleic Acids Res* 36: e126.

8. Purcell S, Neale B, Todd-Brown K, Thomas L, Ferreira MAR, Bender D, *et al.* (2007): PLINK: a tool set for whole-genome association and population-based linkage analyses. *Am J Med Genet* 81: 559–575.

9. Kirov G (2010): The role of copy number variation in schizophrenia. *Expert Rev Neurother* 10: 25–32.
